# The Meniscus Tear: A Review of Stem Cell Therapies

**DOI:** 10.3390/cells9010092

**Published:** 2019-12-30

**Authors:** George Jacob, Kazunori Shimomura, Aaron J. Krych, Norimasa Nakamura

**Affiliations:** 1 Department and Orthopaedic Surgery, Osaka University Graduate School of Medicine, Osaka 565-0871, Japan; drgeorge.jac@gmail.com (G.J.); kazunori-shimomura@umin.net (K.S.); 2 Department of Orthopaedic Surgery, Mayo Clinic, Rochester, MN 55905, USA; 3 Institute for Medical Science in Sports, Osaka Health Science University, Osaka 530-0043, Japan; 4 Global Centre for Medical Engineering and Informatics, Osaka University, Osaka 565-0871, Japan

**Keywords:** meniscus, stem cell, tissue engineering, meniscal regeneration, meniscal repair

## Abstract

Meniscal injuries have posed a challenging problem for many years, especially considering that historically the meniscus was considered to be a structure with no important role in the knee joint. This led to earlier treatments aiming at the removal of the entire structure in a procedure known as a meniscectomy. However, with the current understanding of the function and roles of the meniscus, meniscectomy has been identified to accelerate joint degradation significantly and is no longer a preferred treatment option in meniscal tears. Current therapies are now focused to regenerate, repair, or replace the injured meniscus to restore its native function. Repairs have improved in technique and materials over time, with various implant devices being utilized and developed. More recently, strategies have applied stem cells, tissue engineering, and their combination to potentiate healing to achieve superior quality repair tissue and retard the joint degeneration associated with an injured or inadequately functioning meniscus. Accordingly, the purpose of this current review is to summarize the current available pre-clinical and clinical literature using stem cells and tissue engineering for meniscal repair and regeneration.

## 1. Introduction

The meniscus is an essential member of the knee joint and without its proper functioning, pathologic force distribution and instability occur in the knee, negatively affecting overall joint biomechanics [1,2]. Due to the avascular and hypocellular nature of meniscal tissue, it possesses a capacity for healing once damaged [3,4]. Though the guarded prognosis of meniscectomy was noticed as early as 1923 [5], many surgeons still perform total or partial removal of the meniscus to address meniscal tears. This management option remains popular as modern-day medicine is yet to find an effective evidence-based solution. It is now generally accepted that every effort should be made to repair and retain as much native meniscal tissue as possible [6]. This change in approach has led to the introduction of several novel reparative techniques and strategies to restore meniscal function in individuals with meniscal injuries. Owing to the complex phenotype of meniscal tissue, tissue regeneration using stem cell therapy may hold the key to tackling meniscal tears. The use of both meniscal cells and mesenchymal stem cells (MSCs) have proven effective in regenerating meniscal tissue, however meniscal cell harvest poses an unacceptable donor site morbidity and tear site cells have little to no chondrogenic potential [7]. Therefore, the majority of research has been focused on stem cells where there is a reasonable amount of pre-clinical data but limited clinical data.

Meniscal replacement strategies in the form of a collagen meniscal implant and a polyurethane polymer scaffold have been employed over recent years with promising clinical results. However, these implants have been unsuccessful in emulating normal meniscal biomechanics and radiological follow-up does not demonstrate images resembling that of normal meniscus [8,9,10,11]. Meniscal allograft transplantation has also been an option in selected healthcare systems but has several limitations ranging from graft availability, congruence, biocompatibility, fixation problems, and potential infection [8]. With the current preferred management option being meniscal repair, the purpose of this article is to comprehensively review the current status of stem cell treatments in both pre-clinical and clinical studies, dividing them into injection-based and tissue-engineered cell therapies.

## 2. Anatomy of the Meniscus

**Figure 1:**
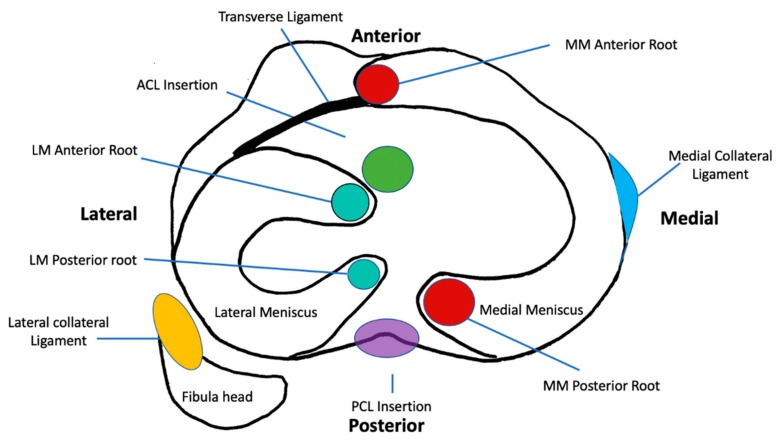
Schematic diagram of the axial section at the level of the tibial plateau depicting the anatomy, attachments, and relations of the menisci. MM, Medial Meniscus; PCL, Posterior Cruciate Ligament; LM, Lateral Meniscus; ACL, Anterior Cruciate Ligament.

The menisci are composed of two semilunar shaped structures divided into a medial and lateral component of which both are biphasic and fibrocartilaginous. They are composed of a dense extracellular matrix (ECM) with low cellularity and vascular supply exclusively to the outer 10–15% of tissue [12,13,14,15]. The menisci are attached to the tibial plateau at anterior and posterior roots and are part of a meniscal ligament complex consisting of the medial collateral ligament (MCL), the transverse ligament, the meniscotibial and meniscofemoral ligaments [12,16,17] (Figure 1).

**Figure 2:**
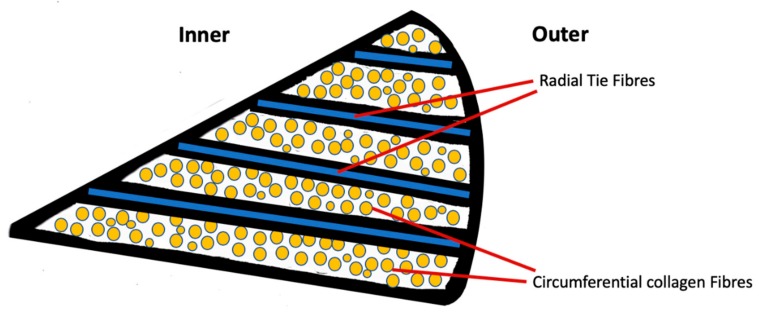
Schematic cross-sectional diagram of the meniscus displaying the circumferential arrangement of collagen fibers and radial tie fibers.

Within the meniscus fibrils, fibers, and fascicles are arranged in diverse patterns depending on the region of tissue [18]. The innermost region consists of small unorganized woven radial collagen fibrils with a structure similar to that of cartilage [19,20], with greater proteoglycan content. The outer region consists of intertwined collagen fibrils in a circumferential orientation with radially oriented three-dimensional arrays of fibers known as “tie-fibers” (Figure 2). They lie perpendicular to the circumferential collagen fibers and originate from the joint capsule creating a complex honeycomb network [18,21]. The root attachments of the meniscus to the tibia are more ligamentous-like structures with a fibrocartilaginous enthesis [22].

The biochemical composition of meniscal tissue is 72% water, 22% collagen, 0.8% glycosaminoglycan (GAG) and 0.012% DNA [23]. Within the meniscal ECM, there is a greater amount of collagen I in the outer red–red zone and a greater amount of collagen II in the inner white–white zone [24]. Numerous proteoglycans exist within meniscal tissue of which the most abundant is aggrecan, others include biglycan, decorin, fibromodulin, lubricin, and elastin [25,26,27]. These proteoglycans provide the meniscus with its viscoelastic, low friction, yet strong phenotype.

The cellularity of meniscal tissue is composed of oval fibrochondrocytes and spindle-shaped fibroblast-like meniscus cells near the outer region connected via long cell extensions [12,28,29]. The cells present in the inner region of the meniscus are chondrocyte-like and are more rounded and embedded within the ECM [30]. Within the superficial zone of the meniscus, one more population of cells described has a flattened, fusiform morphology without any cell extensions. These have been postulated to be progenitor cells with regenerative capabilities [31].

## 3. Functions of the Meniscus

The meniscus plays an important role in normal knee joint mechanics and function by the transmission of joint reaction forces, lubrication, nutrition to the cartilage and shock absorption [32,33]. During standard weight-bearing, the forces applied to the meniscus are known as “hoop stresses”. These are circumferential forces generated as a result of vertical axial forces being converted to horizontal tensile forces owing to the meniscal tissues circumferential collagen fiber arrangement [34]. Shear forces are similarly developed between collagen fibers when the meniscus undergoes radial deformation [35]. The wedge shape of the meniscus allows for better articulation and stability for the rounded femoral condyle on the flat tibial plateau [2,36]. The medial meniscus has also been demonstrated to have a considerable contribution to preventing anterior tibial translation alongside the anterior cruciate ligament (ACL) [37].

It is hypothesized that through a system of micro canals within the meniscal tissue there is the transport of synovial fluid in order to nourish the articular cartilage by compressing synovial fluid into the cartilage reducing friction on the chondral surface [38,39]. Another key feature of the meniscus is the presence of proprioceptive mechanical receptors, in the form of Pacinian corpuscles and Ruffini endings located in the anterior and posterior horns of the menisci contributing to joint position sense and afferent sensory feedback [40,41,42].

It is important to note the several roles of the meniscus and focus on interventions restoring it to full capacity. It can be certainly agreed that the complex phenotype of the meniscus is in accordance with its complex functionality.

## 4. Meniscus Pathology

Meniscal injuries may be acute or degenerative and be as a result of macro-trauma or chronic repetitive attrition commonly encountered in middle-aged and older patients. Acute tears are usually in association with a traumatic event where a combination of compressive, shear, and rotational forces are applied across the meniscus from the femoral condyles onto the tibial plateau. Acute tears are classified into different patterns: Longitudinal, radial, and horizontal, these can progress to more complex tears. In certain situations, tears may displace the tissue and it may get lodged between the femoral condyles, thereby locking the knee joint in flexion. Degenerative meniscal lesions occur more gradually over time and are usually associated with osteoarthritis (OA) [43,44]. Data suggests that the incidence of degenerative tears is higher than earlier believed, as many tears remain asymptomatic [45,46,47]. Degenerative tears are more frequently located in the posterior horn of the medial meniscus and are of horizontal-cleavage or flap tears with some element of tissue destruction [48]. Besides tear morphology, the overall position of the meniscus is important to evaluate. Extrusion of the meniscus can occur concomitantly with certain tear types, particularly root and radial tears, and usually occurs in degenerative lesions in the setting of OA [49,50,51].

**Figure 3:**
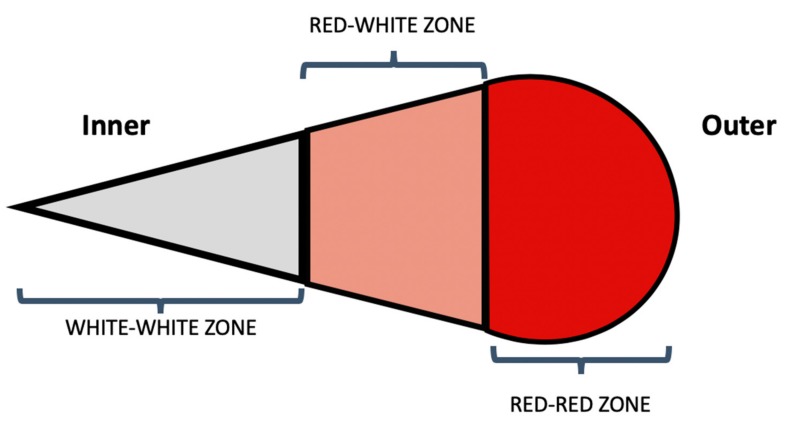
Schematic cross-sectional diagram of the body of the meniscus representing the vascular zones of the meniscus.

The healing potential of a meniscal tear is largely dictated by the tear location. The meniscus has been described to have an inner white–white avascular zone and an outer red-red vascular zone. Between these is a red–white zone of less, but still some degree of, vascularity (Figure 3). Tears involving the inner zone have the least healing potential due to a lack of blood supply [3,52].

When the meniscal function is compromised in the event of an injury, the biomechanics of the knee is deranged. There is increased stress on the cartilage in the joint which can lead to cartilage loss, bony changes, and OA progression [53,54,55,56]. Studies have even shown trabecular bone variations as a result of the loss of meniscal function. In settings of meniscal extrusion, the meniscus no longer absorbs hoops stresses, joint space is reduced and there is an increased possibility in the occurrence of bone marrow lesions [57,58].

## 5. Types of Mesenchymal Stem Cells

Stem cell therapies in musculoskeletal medicine have employed numerous sources of stem cells, and more recently the breakthrough of the induced pluripotent cell has meant cells can now be reprogrammed to perform as stem cells [59]. Treatment strategy focus has been primarily on cartilage, meniscus, and bone to treat chondral defects, meniscal injuries, and fractures. MSCs have been of keen interest in stem cell treatments due to their ease of availability and differentiation capabilities [60]. Cell source is an important consideration for successful outcomes in stem cell therapies [61] and common sources include bone marrow [62], adipose [63], synovium [64], and blood [65]. There is no absolute best cell source as each source has its advantages, disadvantages, and differentiation capacities. Table 1 has been constructed based on studies by Sakaguchi et al. on human MSCs [64] and Yoshimura et al. on rat MSCs [66]. Both studies concluded that synovial tissue was the superior choice of tissue when comparing osteogenic, chondrogenic and adipogenic capacities of the three cell sources. Additional literature has also found synovium to be a superior and effective cell source of MSCs [61,67,68]. Bone marrow has been a popular cell source in the majority of studies. The principal difficulties associated with bone marrow MSCs (BMMSC) is the harvesting process being painful, and their limited differentiation potential with in vitro expansion [69]. Adipose-derived stem cells (ADSCs) have gained popularity for their high yield [70] and ease to procure through liposuction. Literature does, however, report ADSCs to be inferior to synovial MSCs in terms of their chondrogenic and osteogenic differentiation capacities [64]. The clinical advantages and disadvantages of the discussed cell sources have also been outlined in Table 1 [64,69,70,71]. Concerning meniscal tissue, the ideal cell source remains to determined and despite showing varying differentiation capacities between sources in different models the literature still lacks evidence to state one cell source superior to another in meniscal regeneration. 

## 6. Mechanism of Meniscal Repair

The precise mechanism by which a meniscal regeneration occurs remains unknown, it is, however, thought to occur via both extrinsic and intrinsic pathways [72,73,74]. The extrinsic pathway is dependent on the tear site vascularity, where undifferentiated MSCs and growth factors can encourage the repair. The more direct intrinsic pathway occurs via the self-healing capability of the meniscal tissue and is not always a strong contributor to repair [75]. It is known that after meniscal injury the number of MSCs in the synovial fluid increases providing endogenous cells required for repair [76].

As with all healing, angiogenesis is a vital factor in meniscal tear repair too, promoting repair by supplying growth factors and inflammatory processes. The significance of angiogenesis has been demonstrated in a rabbit meniscal defect model where angiogenin treated defects had significantly better healing rates than the control group [77]. This is following other studies that have shown good healing rates in the vascular rich red-red zone of the meniscus [78,79]. Some literature has also shown synovium to contribute some element of vascularity to injured meniscal sites [80,81]. The role of growth factors remains dependent on the injury site vascularity and their anabolic effects have shown to improve MSC differentiation and phenotype [52]. Such growth factors are secreted as a result of the paracrine functions of MSCs into exosomes, allowing them to modulate angiogenesis, cell migration, differentiation, and numerous additional processes [82]. For this reason, research has focused on the application of various growth factors within scaffolds, to meniscal injury models with the hope of an enhanced healing response. Of note, recently transforming growth factor (TGF-β3) and connective tissue growth factor (CTGF) have shown positive results in ovine model meniscal repairs, with the ability to induce cell differentiation towards native zone-specific matrix phenotypes [83,84]. This highlights the key roles of MSCs and growth factors in successful meniscal healing to generate cellular phenotypes resembling that of normal meniscal tissue.

Mechanical factors also affect meniscal healing considerably and can have undesirable effects on healing when the meniscus is loaded pathologically. This is the rationale behind stabilizing tears with sutures to immobilize tear sites. Though tear site stability seems to be more important than complete immobilization of the joint [85,86]. Normal physiologic loading of the meniscus has been shown to have anti-inflammatory and overall anabolic effects while pathological loading has the exact opposite effect increasing catabolism, inflammation, and cell death [87]. Overall it can be summarized that meniscal repair is very much dependent on vascularity and stability of the tear site. Good vascularity facilitates pluripotent stem cells and endogenous growth factors to interact and mediate the production of repair meniscal tissue.

## 7. Pre-Clinical Studies

### 7.1. Stem Cell Injection

Simple MSC injections from different sources have been employed in various animal models to evaluate their effects on tissue regeneration and healing. Recently, synovium has been identified as a good source of MSCs as these cells have a high potential for proliferation and chondrogenic differentiation [64,88,89]. Nakagawa et al. [90] combined allogeneic synovial MSCs and a suture repair to a meniscal defect model in a porcine model. In their study, the time to outcome assessment was only 12 weeks, though they reported superior results than in an isolated suture repair group. The MSC group demonstrated higher histology scores, collagen deposition and greater tensile strength in the repair site. They noted no immunologic reactions despite not using any immunosuppressive drugs in the subjects. A similar study employing allogeneic synovial MSCs was performed by Hatsushika et al. [91] using multiple doses of intraarticular synovial MSC injections in a porcine model. The defect model was somewhat large where the entire anterior half of the medial meniscus was removed. Subjects injected with MSCs showed defect filling with synovial tissue at 2 weeks. At 16 weeks when compared to the control group, the MSC group had superior quality tissue with improved safranin-o and collagen I and II staining. They concluded that synovial derived MSCs promoted meniscal regeneration and were more effective with repeated intraarticular injection use, though the optimal number of injections was yet to be determined. Both studies [91,92] did also mention that this was an acute meniscal tear model and that regenerative results may be different in a chronic scenario as demonstrated by Ruiz-Iban et al. [92]. In this study, rabbit meniscal lesions were created, and some subjects underwent an acute treatment protocol while others were treated after 3 weeks to simulate tear chronicity. Meniscal healing was significantly better in the acutely treated groups, thereby confirming tear chronicity having a role to play in tissue healing.

A study by Ferris et al. [93] administered an intraarticular injection of autologous BMMSCs to horse stifle joints after the diagnosis of a meniscal tear by arthroscopy. This model is more accurate in that the time from injury to injection simulated that of a normal clinical scenario as opposed to the creation of a defect and immediate subsequent treatment. The subjects received only debridement followed by the intraarticular BMMSC injection, no suture repair was performed alongside the treatment. Eighteen out of twenty-four horses with documented meniscal lesions returned to work with 9 horses reaching their previous levels of activity. The outcomes of this study were compared to previous reports and they reported significant positive outcomes in BMMSC injections for the treatment of meniscal lesions in horses. Another study employing only autologous BMMSCs in a canine meniscus tear model found better healing responses in injected subjects compared to the controls [94]. Injected subjects exhibited significantly better histology with marked angiogenesis, fibroblast proliferation, chondrogenesis, and collagen deposition. They concluded that BMMSCs were effective in regenerating meniscus tissue and could function by either BMMSC differentiation or mediator release signaling a healing mechanism. From these models, it is evident that MSCs have a role to play in meniscal regeneration and that subjects who received some form of stem cell injection, whether combined with a repair or not, did display superior healing responses and histology. 

As mentioned earlier, it has still not been determined which cell source is superior for MSC treatments, but each has shown promising results with their own advantages and disadvantages. In addition, allogeneic cells have also shown promising results, which would have its own benefits for manufacturing, cost, and development of single-stage treatment strategies. Table 2 summarizes pre-clinical stem cell injection data studies included in our review. 

### 7.2. Tissue Engineering

Tissue engineering techniques concerning meniscus regeneration have employed MSCs in combination with engineered scaffolds and growth factors to achieve more efficient and better-quality repair/regenerate tissue. Several scaffolds have been developed ranging from synthetic polymers to more natural tissue-derived sources. In vitro studies have demonstrated the outcomes of using different cell types [7] and the benefits of adding growth factors to cultures that can promote GAG production and improve cell differentiation enhancing the bioactivity of the overall scaffold for integration [95]. Such study models have also shed light on the effects of inhibitory effects on the meniscal repair by interleukin-1 and tumor necrosis factor-α commonly upregulated in injured joints [96,97,98]. The mechanical variations of the inner and outer zone cells of the meniscus have also been thought to bring about variation in gene expression and protein regulation [99]. The challenge in scaffold optimization is in finding a delicate balance between mechanical strength and bioactivity.

Zhang et al. [100] in a goat model demonstrated the healing capacity of BMMSCs transfected with human insulin-like growth factor 1 (hIGF-1) using a calcium alginate gel for delivery into a full-thickness meniscal defect. Their study included three control groups: one group with cells and no hIGF-1 transfection, one with the alginate gel alone, and one without any form of treatment. The group repaired with hIGF-1 transfected cells developed the best reparative tissue with margins difficult to delineate from the neighboring native tissue. This group also demonstrated a greater number of cells, more cartilaginous tissue, and higher GAG content than the control groups. This is a clear example of an in vivo application of a growth factor (hIGF-1) in combination with MSCs promoting and enhancing the meniscal regenerate.

Moriguchi et al. developed a natural tissue-engineered construct (TEC) consisting of a high- density monolayer culture of allogeneic synovial MSCs in the presence of ascorbic acid [101]. Four-millimeter cylindrical defects were created in a porcine meniscus model and repaired using TEC or left untreated for control. All TEC implanted defects were filled with well-integrated repair tissue, while the controls remained empty or partially filled. Histology of the TEC repair displayed cartilage-like cells with lacunae indicative of fibro-cartilaginous tissue. The incidence of chondral injury 6 months after defect creation was significantly less in the TEC group in comparison to the control. Interestingly, TEC is scaffold-free and yet provides good bioactivity and mechanical support to the repair site. The study concluded that a fibrocartilaginous repair tissue is a desirable result for meniscal defects as meniscal tissue displays mixed characteristics of hyaline cartilage and fibrous tissue. This animal study validated that TEC could be an effective solution to achieve the desirable hybrid tissue qualities required to fill a meniscal defect and retard OA progression that typically follows a meniscal injury. Kondo et al. [102] used autologous synovial MSC aggregates to repair meniscal defects in a primate model. They found the medial meniscus in the repair group to have larger regenerate at both time points of 8 and 16 weeks. The regenerated meniscus also stained better with safranin-o and had T1rho magnetic resonance imaging (MRI) that resembled native meniscal tissue. Both the control and the study group did show OA changes, though the MSC treated group had better scores. This study again demonstrated both the regenerative potential of synovial MSCs and also the use of aggregates alone without the need for a fixation method to treat meniscal injury. 

Desando et al. [103] used a hyaluronic acid (HA) scaffold seeded with autologous BMMSCs in an ovine meniscal defect model. No fixation technique was employed as the mesh had intrinsic adhesive properties [104]. The BMMSC seeded HA scaffold group revealed a superior repair with a smooth restored surface and good proteoglycan content compared to the control group. There was greater expression of collagen type I and II and lower expression of matrix metalloproteinase-13 and interleukin 1 beta indicating less collagen degradation [105,106,107]. The bone marrow seeded HA group was therefore determined to be more chondroprotective when compared to the control group. This study confirmed that BMMSCs can enhance a more effective meniscal repair, as well as reduce the biochemical changes associated with the progression of OA. Table 3 summarizes the pre-clinical tissue engineered stem cell studies included in our review. 

## 8. Clinical Studies

### 8.1. Stem Cell Injection

Clinical studies evaluating the effects of MSC injections in the knee joint are limited, but early clinical data suggests encouraging results. Currently, there have not been any reported safety concerns or side-effects in the clinical use of MSC injections.

There is only one randomized double-blind controlled study to date studying the effects of MSC injections into the knee post medial meniscectomy [108]. The study contained 55 subjects in 3 groups who underwent a percutaneous injection of allogeneic MSCs with one group receiving 50 × 10^6^ cells another 150 × 10^6^ cells and control receiving only HA. At 12 months follow up, MRI scan findings reported a significant increase in meniscal volume in 24% of patients receiving 50 × 10^6^ cells and 6% receiving 150 × 10^6^ cells. None of the control group patients demonstrated an increase in meniscal volume. The study is limited to MRI scan being the only objective outcome measure, but the study methodology is rigorous in that it has the advantage of being blinded and randomized.

Pak et al. [109] reported the results of a single patient who received an ultrasound guided autologous adipose stem cell (ASC) intraarticular injection to the knee joint for treatment of an isolated meniscus tear. The final injection mixture contained the ASCs, platelet-rich plasma (PRP), HA and calcium chloride. This patient also received follow up injections of PRP, HA, and dexamethasone. The patient was followed up for a period of 18 months and reported continued improvement in knee pain scores and superior knee function. A 3-month MRI scan reported almost complete resolution of the meniscal tear. Radiologic evaluation beyond this time point was not available. This study lacked control therefore, it remains difficult to determine the efficacy of this treatment. However, it is worth noting the same patient did undergo PRP and HA injections prior to the stem cell injection and had reported unsatisfactory outcomes with these treatments. Centeno et al. [110] reported a patient who received an intraarticular injection of BMMSCs which were expanded using platelet lysate extracted from the patient’s own blood. After the radiologic MRI diagnosis of degenerative changes in the medial meniscus and medial femoral condyle, the patient was injected with the BMMSCs after expansion in the growth factors present in the platelet lysate. The expanded MSCs were injected into the knee joint along with fresh whole marrow. The patient did also receive 2 subsequent injections of platelet lysate combined with 1ng/mL dexamethasone. The post-procedure 3-month MRI scan showed evidence of increased meniscus volume, and the patient did report improved pain scores. This is encouraging data for a relatively simple procedure, though this case report is limited to one patient without a tissue biopsy, so the exact nature of the regenerated tissue remains unknown.

A recent paper by Onoi et al. [111] reported second-look knee arthroscopy findings in two patients after ASC injections. However, only one of the treated patients had a second-look arthroscopy. This patient underwent a partial meniscectomy for a degenerative tear in the posterior horn of the medial meniscus. At 6 months after ASC injection, the second-look arthroscopy not only showed improved cartilage status but also repair of the resected part of the meniscus. The meniscus tissue was not biopsied, and the sample size was small, but the second-look arthroscopy findings were encouraging. 

Sekiya et al. [112] studied the addition of synovial MSCs to degenerative medial meniscus lesions in 5 patients. The patients underwent an initial arthroscopy where the lesions were confirmed, repaired with sutures and finally, a synovial tissue biopsy was performed. The synovial tissue was cultured and expanded for 14 days and a repeat arthroscopy was performed where a synovial MSC cell suspension was delivered to the site of the repair. Patients reported significant clinical score improvement by 2 years and 3D MRI scan results reported no evidence of tear at the repair site. Table 4 summarizes the clinical stem cell injection studies found in the literature. 

### 8.2. Tissue Engineering

The combination of MSCs and tissue engineering is also an emerging field from a clinical standpoint, and a greater number of studies have focused more on finding a solution for cartilage defects. Whitehouse et al. [113] reported a case series of 5 patients where BMMSCs were injected onto a collagen scaffold and sutured into an avascular meniscal tear using vertical mattress sutures. Three out of 5 patients reported positive outcomes beyond 12 months with significantly improved clinical scores and subsequent MRI scans showing in situ repair along with a reduction in the abnormal signal of the scaffold. Two patients had a failure of treatment, sustaining repeat tears at around 15 months. A very recent study by Olivios-Meza et al. [114] combined a polyurethane meniscal scaffold with MSCs for meniscal repair. They divided patients into two groups, one with an acellular scaffold repair and another enriched with MSCs. Scaffolds were arthroscopically implanted into patients who had a history of receiving a meniscectomy in the past. MSCs were obtained from a blood draw after the subjects received 3 days of consecutive 300 μg subcutaneous G-CSF injections in order to increase the peripheral bloodstream MSC pool. After cell separation, CD90+ expression cells were isolated, cultured and seeded on the scaffold. Scaffolds were sutured to the neighboring meniscal tissue and joint capsule to fill the defect with all inside sutures. Outcomes were determined by assessing cartilage adjacent to the repair site with an MRI cartigram. They noted a significant radiologic and clinical improvement in both groups but concluded the addition of MSC to the polyurethane scaffold repair made no difference. This study did, however, have a small sample size with no randomization, and the post-operative MRI scan findings of the meniscal repair site were not reported. Table 5. summarizes the clinical studies available on tissue-engineered meniscal tear treatments. 

## 9. Conclusions

In summary, the available literature demonstrates that MSCs appear to be safe and effective in producing superior quality meniscal repairs. There is compelling pre-clinical data that has studied the effects of various cell sources, scaffolds, and even growth factor additives. Despite this, presently there is no consensus on the ideal cell source and scaffold for meniscus regeneration. Current limitations of the data include a lack of long-term follow-up, control groups, and objective outcome endpoints. In comparison to articular cartilage regeneration, where there have been more clinical studies that have reported on repair tissue histology, second-look arthroscopy, and radiologic imaging, robust outcomes are still lacking for meniscal stem cell therapy studies. 

At the end of our review, we do note that each therapy and mode of delivery has its own advantages and disadvantages and at present, we cannot identify or recommend a certain intervention as a standard of care. We do however encourage the use of stem cell therapies as an investigational agent in the setting of meniscal injuries in order to increase the available literature and evidence for or against its use.

The solution to meniscal tissue regeneration is a particularly elusive one and appears far more complex than that of cartilage regeneration due to the complex phenotype and function of meniscal tissue. We anticipate that stem cell therapies will become more effective in the near future in order to aid meniscal repair modalities, thereby adding another weapon to retard dreaded OA progression in the knee. 

## Figures and Tables

**Table 1:** Summary table showing differentiation capacities as well as advantages and disadvantages of bone marrow, adipose and synovium mesenchymal stem cells (MSCs) [64,66,67,68,69,70,71].

MSC Source	Osteogenic	Chondrogenic	Adipogenic	Clinical Advantage	Clinical Disadvantage
Bone marrow	+++	+++	++	Aspiration can be done under L/A	Invasive, Painful, Low yield
Adipose	+	+	+++	Less painful than marrow aspiration and high yield	L/A toxic to ASCs therefore harvest preferable under GA
Synovium	+++	+++	+++	Painless, Minimally invasive and Minimal tissue requirement	Staged surgery, cells require expansion

Abbreviations: L/A, Local anesthesia.

**Table 2:** Summary of pre-clinical studies using stem cell injections.

Author/Year	Animal/Defect Model	Source/Cell number/Method of Delivery	Control	Outcome Measurement/Timeline	Results
Nakagawa et al. [90]/2015	Micro minipig/Medial Meniscal full thickness longitudinal tear	Allogeneic Synovial MSCs/20 × 10^6/^Suture repair +MSC suspension injection	Suture repair + Acellular suspension	Macro and Histo analysis, IMHC, TEM, MRI, Biomechanical analysis/12 weeks	Macroscopy: Scores were better in MSC group at all time points compared to control. Histology: Scores were higher in MSC group at all time points compared to control. TEM: dense collagen fibrils in MSC group, none in control. MRI: MSCs group has T1rho values closer to intact meniscus than control. Higher tensile strength in MSC group
Hatsushika et al. [91]/2014	Pig/Medial meniscus anterior half resection	Allogeneic Synovial MSCs/50 × 10^6^ × 3/IA injections x3 with 2-week gaps of synovial MSCs	PBS injection	Macro and Histo analysis. IMHC TEM MRI/16 weeks	Macro: regeneration of anterior medial meniscus in both groups. Histo: better Safranin-O staining in MSC group, COL I and II staining showed larger representation in MSC group. Mod Pauli’s score was higher in MSC group. MRI: regenerate area appeared more organized in MSC group
Ferris et al. [93]/2014	Horse/Meniscal tear	Autologous BMSCs/15–20 × 10^6/^Arthroscopy + IA injection of BMSCs	Previous surgical data	Return to work/24 months	18/24 (75%) horses with meniscal lesions returned to work. 9 returned to previous level of activity
Abdel-Hamid et al. [94]/2005	Dog/Longitudinal full thickness meniscal tear	Autologous BMSCs/2-4ml aspirate/Injection at tear site	Tear with no treatment	Histo, IMHC/12 weeks	Better healing response in injected group compared to control. Histo: angiogenesis, collagen deposition and fibroblast proliferation in injected compared to control

Abbreviations: Macro, Macroscopic; Histo, Histology; IMHC, Immunohistochemistry; TEM, transmission electron microscopy; MRI, magnetic resonance imaging; BMMSC, bone marrow MSCs.

**Table 3:** Summary of pre-clinical studies using tissue engineering.

Author/Year	Animal/Defect Model	Source/Cell number/Method of Delivery	Control	Outcome Measurement/Timeline	Results
Zhang et al. [100]/2009	Goat/full thickness defect in medial meniscus anterior horn	BMMSC with transfection of hIGF-1/30 × 10^6^/mL/Calcium alginate gel into defect	Defect with nil treatment	Histo, TEM, GAG Assay MRI/16 weeks	BMMSC w/hIGF-1 group had better repair tissue without clear margin. Large number of well aligned cells within repair defect. TEM showed round oval like chondrocyte like cells. MRI: smooth continuous anterior horn Higher GAG content to control
Moriguchi et al. [101]/2013	Pig/4 mm cylindrical defect in medial meniscus	Synovial MSC/0.2 × 10^6^ cells—3 weeks culture/3D matrix construct (TEC)	Nil treatment	Gross morphology Histo/6 months	TEC implanted defects showed fibrocartilaginous repair and integration compared to control. Histo: cartilage like cells with nuclei in lacuna
Kondo et al. [102]/2017	Monkey/Anterior horn of medial meniscus Partial Meniscectomy	Synovial MSCs/0.25 × 10^6^/Aggregates	Nil aggerate	Macro and Histo analysis MRI/8 weeks (n = 3) 16 weeks (n = 4)	Macro: Regeneration in control and MSCs groups with MSC showed larger medial meniscus at 8 and 16 weeks. Histo: Safranin-O slight staining at 8 weeks, positive at 16 weeks. No staining in control MRI: MSC groups closer resembled intact menisci compared to control.
Desando et al. [103]/2016	Sheep/Unilateral medial meniscectomy	Bone marrow concentrate or BMSCs/BMC:39 × 10^6^ BMSCs: 6 × 10^6^/Arthrotomy Bone marrow or BMMSC in HA mesh	Nil treatment	Gross morphology Microtomography Histo Immunohistology/12 weeks	Meniscal tissue regeneration greatest in BMC + HA group. Both BMC and BMSCs group showed good cell density and proteoglycan content compared to control. BMC+ HA group had higher expression of Col II than I compared to BMSCs group.

Abbreviations: BMMSCs, Bone marrow mesenchymal stem cell; Macro, Macroscopic; Histo, Histology; TEM, Transmission electron microscopy; MRI, Magnetic resonance imaging; GAG, Glycosaminoglycan; MSC, Mesenchymal stem cell; HA, Hyaluronic Acid.

**Table 4:** Summary of clinical studies using stem cell injections.

Author/Year	Study type/Patient number	Source/Cell Number	Method of Delivery	Outcome/Follow Up	Results
Vangsness et al. [108]/2014	Randomized control trial/55	Allogeneic MSCs derived from BMAC/A:50 × 10^6^ B:150 × 10^6^	Percutaneous knee injection	MRI VAS Lysholm knee score/2 years	Significant improvement in scores at 3 months. 12-month MRI at 12 months: significant increase in meniscal volume in MSC groups compared to control
Pak et al. [109]/2014	Case control/1	Abdominal liposuction/Not reported	Percutaneous knee injection	VAS, Functional rating index, ROM, MRI/18 months	At 3 months MRI showed no evidence of meniscal tear, Symptoms improved and asymptomatic at 18 months
Centeno CJ et al. [110]/2008	Case control/1	Iliac crest BMAC/45.6 × 10^6^	Percutaneous knee injection	VAS, Functional rating index, MRI/3 months	Increased meniscus volume on MRI. Decreased VAS Score from 3.33 to 0.13
Onoi et al. [111]/2019	Case report/2	Liposuction from thigh/5.5 × 10^6^	Percutaneous knee injection	MRI KOOS Arthroscopy/6 months	Both patients reported better scores at 6 months follow up. 2nd look arthroscopy showed meniscal tear healing
Sekiya et al. [112]/2019	Case series/5	Arthroscopically harvested Synovial Tissue/32–70 × 10^6^	Arthroscopic transplantation of autologous synovial MSC suspension to sutured meniscal lesion	Lysholm knee score KOOS NRS 3D MRI/24 months	Significant improvement of Lysholm score by 1 year. Other scores significantly increased by 2 years 3D MRI: Tears were indistinguishable

Abbreviations: MSC, Mesenchymal stem cell; BMAC, Bone marrow aspirate concentrate; Macro, Macroscopic; Histo, Histology; MRI, Magnetic resonance imaging; VAS, Visual Analogue Score; ROM, Range of motion; KOOS, Knee Injury and Osteoarthritis Score; NRS, Numeric Rating Scale.

**Table 5:** Summary of clinical studies using tissue engineering.

Author/Year	Study Type/Patient Number	Source/Cell Number	Method of Delivery	Outcome/Follow Up	Results
Whitehouse et al. [113]/2017	Case Series/5	Iliac crest BMAC/1 × 10^6^/cm^2^	Arthroscopic MSC injection into Collagen Scaffold	IKDC Score Lysholm Score. ROM MRI/2 years	3 patients reported significantly improved clinical outcomes and MRI imaging
Olivos-Meza [114]/2019	Case Series/17	s/c G-CSF x 3 blood draws. Cell separation isolation and culture CD 90+ cells/20 × 10^6^	Arthroscopic implantation of MSC cell seeded polyurethane scaffold vs. acellular polyurethane scaffold	Lysholm Score MRI/12 months	Both groups improved in Lysholm scores. No intergroup difference was noted. MRI Cartigram: Improved in cell seeded scaffold at 9 months but reduced to initial value at 12 months

Abbreviations: BMAC, Bone Marrow aspirate concentrate; MSC, Mesenchymal stem cell; IKDC, International Knee Documentation Committee; ROM, Range of motion; MRI, Magnetic resonance imaging; G-CSF, Granulocyte Colony stimulating factor.
